# Supplementary material for: The interactome of intact mitochondria by cross-linking mass spectrometry provides evidence for coexisting respiratory supercomplexes
Source: Mol Cell Proteomics. 2017 Dec 8;17(2):216–32. doi: 10.1074/mcp.RA117.000470 (PMC5795388; doi:10.1074/mcp.RA117.000470)
Supplement: Supplemental Data [file supp_RA117.000470_133922_0_supp_23978_fzffwf.zip › spectra_annotation/mito_spectra_annotation/9-1-3-1-11-1.pdf]

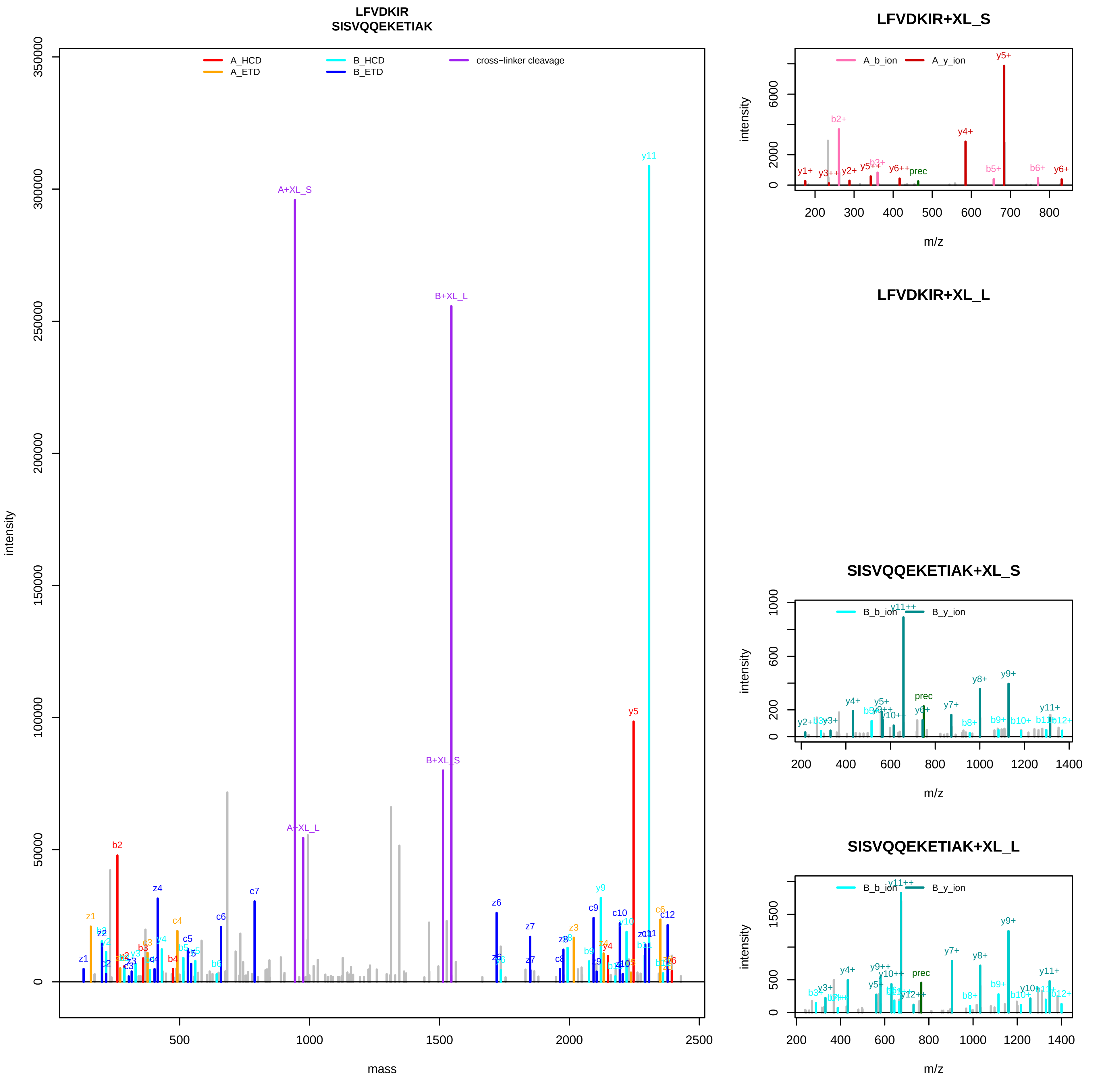
LFVDKIR
SISVQQEKETIAK
A_HCD
A_ETD
B_HCD
B_ETD
cross−linker cleavage
A+XL_S
B+XL_L
B+XL_S
A+XL_L
y11
y5
b2
z4
c7
c6
z6
z7
c9
y9
c10
y10
c12
c6
z1
z2
c4
c5
z3
z8
intensity
mass
0
50000
100000
150000
200000
250000
300000
350000
500
1000
1500
2000
2500
LFVDKIR+XL_S
A_b_ion
A_y_ion
y5+
b2+
y4+
y1+
y3++
y2+
y5++
b3+
y6++
prec
b5+
b6+
y6+
intensity
m/z
0
2000
6000
200
300
400
500
600
700
800
LFVDKIR+XL_L
SISVQQEKETIAK+XL_S
B_b_ion
B_y_ion
y11++
y8+
y9+
prec
y4+
y5+
y7+
y11+
intensity
m/z
0
200
600
1000
200
400
600
800
1000
1200
1400
SISVQQEKETIAK+XL_L
B_b_ion
B_y_ion
y11++
y9+
y7+
y8+
y4+
y9++
y10++
prec
y11+
intensity
m/z
0
500
1500
200
400
600
800
1000
1200
1400
